# Supplementary material for: Netcombin: An algorithm for constructing optimal phylogenetic network from rooted triplets
Source: PLoS One. 2020 Sep 18;15(9):e0227842. doi: 10.1371/journal.pone.0227842 (PMC7500971; doi:10.1371/journal.pone.0227842)
Supplement: S4 File — (ZIP) [file pone.0227842.s004.zip › SequenceData-Triplet/sequenceData-10-20-30-0-Triplet/length 200, numseq 20, rate 10^-7/2 seq.pdf]

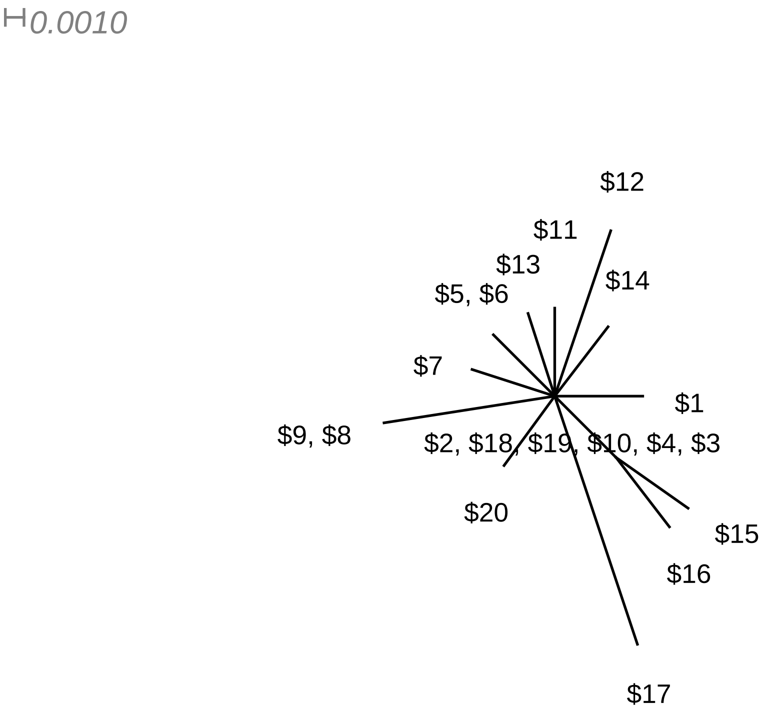
0.0010
$12
$11
$13
$14
$5, $6
$7
$1
$9, $8
$2, $18, $19, $10, $4, $3
$20
$15
$16
$17
